# Theoretical Perspective on an Ideomotor Brain-Computer Interface: Toward a Naturalistic and Non-invasive Brain-Computer Interface Paradigm Based on Action-Effect Representation

**DOI:** 10.3389/fnhum.2021.732764

**Published:** 2021-10-28

**Authors:** Solène Le Bars, Sylvie Chokron, Rodrigo Balp, Khalida Douibi, Florian Waszak

**Affiliations:** ^1^Altran Lab, Capgemini Engineering, Paris, France; ^2^Université de Paris, INCC UMR 8002, CNRS, Paris, France; ^3^Fondation Ophtalmologique Adolphe de Rothschild, Paris, France

**Keywords:** non-invasive brain-computer interface, ideomotor, action-effect prediction, intention decoding, human voluntary action

## Abstract

Recent years have been marked by the fulgurant expansion of non-invasive Brain-Computer Interface (BCI) devices and applications in various contexts (medical, industrial etc.). This technology allows agents “to directly act with thoughts,” bypassing the peripheral motor system. Interestingly, it is worth noting that typical non-invasive BCI paradigms remain distant from neuroscientific models of human voluntary action. Notably, bidirectional links between action and perception are constantly ignored in BCI experiments. In the current perspective article, we proposed an innovative BCI paradigm that is directly inspired by the ideomotor principle, which postulates that voluntary actions are driven by the anticipated representation of forthcoming perceptual effects. We believe that (1) adapting BCI paradigms could allow simple action-effect bindings and consequently action-effect predictions and (2) using neural underpinnings of those action-effect predictions as features of interest in AI methods, could lead to more accurate and naturalistic BCI-mediated actions.

## Introduction

Our ability to interact with our environment seems limitless. We can learn to use keyboards—in the office using 10 fingers, at home using only our thumb on the touchscreen of our phone. We can learn to play violin, to drive a car, to do heart surgery, and so on. According to the ideomotor principle of action control, such intention-based actions are performed to produce internally pre-specified and desired effects in the environment (see James, 1890; Stock and Stock, 2004). In this respect, any motor action would result in, or rather from, anticipating its perceptual consequences (Greenwald, 1970; Le Bars et al., 2016).

Pushing the frontiers of natural motor actions, recent advances in neuroscience and engineering are enabling human beings to directly act upon the environment with “thoughts” through Brain-Computer Interfaces (BCI). In a typical non-invasive BCI system, the user’s neural activity is recorded via brain imaging techniques (e.g., EEG, fNIRS, fMRI), before being decoded with computational and Artificial Intelligence (AI) methods. This last phase allows the translation of the brain signals into digital commands that are understandable by the connected device(s) (e.g., a computer, a robot etc.).

Besides the obvious benefit of BCIs for patients suffering from motor impairments, the dramatic expansion of this technology (see Douibi et al., 2021) raises important questions regarding the *disembodied* nature of resulting actions (Steinert et al., 2019). Notably, one might wonder whether it is even possible to qualify BCI-mediated actions as real human actions, given the potential reduction of sense of agency or responsibility it might cause in users (see Limerick et al., 2014; Rainey et al., 2020). Moreover, it is worth noting that most of non-invasive BCI paradigms aim to enable “acting with thoughts” but do not necessarily respect fundamental aspects of neuroscientific models of human actions, especially regarding the perceptual counterpart of action, which remains barely considered in BCI-mediated actions (see Wang et al., 2019).

In the current article, we attempted to conciliate the neuroscientific models of human actions with non-invasive BCI methods by proposing an innovative and more naturalistic BCI paradigm that would notably take advantage of the ideomotor principle.

In the first section, we summarized the most important evolutions of the ideomotor theory and its alternatives, which all emphasize the importance of action-effect prediction in human action.

In the second section, we reviewed the actual main kinds of non-invasive BCIs and we discussed their limitations in the light of previous motor action models.

Finally, we proposed a new experimental BCI paradigm directly inspired by the ideomotor principle. We believe that (1) adapting BCI paradigms could allow **simple action-effect bindings** and consequently **action-effect predictions** and (2) using neural underpinnings of **those action-effect predictions** as features of interest in Artificial Intelligence (AI) technics, could lead to more accurate and naturalistic BCI-mediated actions.

## The Ideomotor Principle: Origins, Evolutions and Alternatives

Recent decades gave rise to a school of thought, which postulates that, in our brain, perceiving our environment and acting upon it is a unified process. Notably, in line with conditioning theories, the *ideomotor* and *common coding* principles claim that if actions and their effects are repeatedly contingent, actions would end up in being coded in terms of the effects they evoke in the environment (e.g., Prinz, 1997; Hommel et al., 2001; Waszak et al., 2012).

Such sensorimotor contingencies and action-effect mapping could rely on reinforcement learning (e.g., Colzato et al., 2007; Muhle-Karbe and Krebs, 2012) or active inference (e.g., Friston et al., 2016) processes. Indeed, those models notably emphasize on the fact that action-effect bindings are continuously updated through experience.

In the Ideomotor framework, action is thus conceived of as perceptual states. Not present perceptual states, but *future* perceptual states. In other words, a voluntary action is supposed to be primarily driven by the anticipated representation of its expected effect or outcome (see Shin et al., 2010 for a comprehensive review).

**FIGURE 1:**
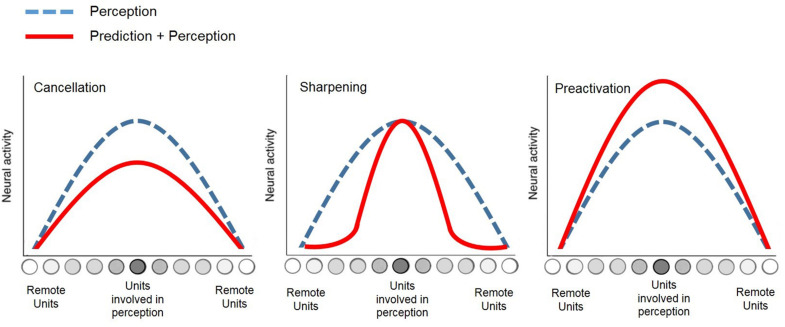
Schematic of neural patterns underlying action-effect prediction relative to perception.

At a neural level, different theories have linked this action-effect prediction to distinct patterns of brain activations such as the *cancelation* (see Wolpert et al., 1995), the *preactivation* (see Waszak et al., 2012), or the *sharpening* (see Kok et al., 2012) of activity in sensory and perceptual brain areas, which are usually related to the action (see Figure 1).

On the one hand, cancelation of activity in expected sensory areas would result from a *forward model* in the motor system that allows the prediction of action-outcomes. In fact, this neural cancelation related to perceptual expectations would keep the agent (i.e., the individual performing the action) maximally sensitive to unexpected or important outcomes, to optimize learning or planning of new actions (see Wolpert et al., 1995; Stanley et al., 2007). The cancelation account perfectly matches with the well-known phenomenon of *sensory attenuation*, which corresponds to a decrease in neural activity (e.g., Baess et al., 2011; Klaffehn et al., 2019) or perception performances (e.g., Cardoso-Leite et al., 2010), and which is commonly associated to the processing of expected outcomes.

On the other hand, the preactivation theory states that action-effect prediction would result from an early enhancement of activity in perceptual areas, which are typically involved in the sensory processing of the action outcome, even though it has not occurred yet in the environment (see Waszak et al., 2012; Roussel et al., 2013, 2014). Previous experiments have successfully demonstrated the existence of neural activation in the sensory units linked to the action, by using brain imaging techniques such as fMRI (e.g., Kühn and Brass, 2010; Kühn et al., 2011) and EEG (e.g., Hughes and Waszak, 2014). Thus, from this point of view, sensory attenuation would result from a more complex discrimination between the observed action-effect and the pre-activation of the predicted effect that occurs before, relative to when no prediction—or misprediction (see Hsu et al., 2015)—is present.

Halfway between the two last principles of *cancelation* and *preactivation*, the *sharpening* account (Kok et al., 2012) argued that sensory expectation would rather result from a suppression of activity in perceptual units that are not supposed to be involved in the processing of forthcoming effects, while neural activity in expected perceptual areas would remain high in parallel. Further experimental evidence of this model was provided recently via fMRI (Yon et al., 2018).

Overall, these neuroscientific theories related to motor action converge toward the two following notions. First, the perceptual component of action is essential and inseparable from motor control, which is notably observable through the action-effect prediction mechanism. Second, the action-effect prediction necessarily relies on the modulation of activity in perceptual brain areas, as synthesized in Figure 1.

However, the precise temporal dynamics of this anticipated action-effect representation is still under debate (see Waszak et al., 2012; Desantis et al., 2014). For instance, some experimental evidence suggested that action-effect representation was activated relatively late, during the action planning step (e.g., Ziessler and Nattkemper, 2011), while other studies—in line with the strong version of the Ideomotor principle—demonstrated that it should occur earlier, during the action selection stage, and before the action initiation (e.g., Paelecke and Kunde, 2007; Le Bars et al., 2016; Dignath et al., 2020).

To our knowledge, no existing BCI paradigm is congruent with the strong Ideomotor view, by using the EEG markers related to the perceptual prediction occurring ***before*** the BCI-mediated action.

## Actual Non-Invasive Brain-Computer Interface Paradigms

Three main kinds of non-invasive BCI paradigms are commonly described in literature, depending on the agent’s task and the patterns of brain activity that are consequently generated (see Kögel et al., 2019). First, *passive BCIs* rely on brain activity that is not voluntarily produced by the user, to monitor his/her neurocognitive or affective state and adequately adapt the environment or issue a warning (e.g., Zander and Krol, 2017). Second, *reactive BCIs* are based on brain activity changes reflecting the agent’s voluntarily focused attention on a specific external stimulus (e.g., Guger et al., 2012). Third, *active BCIs* require the user to apply intentionally a particular mental strategy, such as motor imagery (MI) that usually implies imagining a limb movement without actually performing it (MI-BCI; e.g., Salvaris and Haggard, 2014).

Although these three BCI paradigms eventually lead to outputs or new events in the users’ environment, only reactive and active BCI paradigms allow the agents to *intentionally* perform those changes, by linking a prior intention to act, to a final effect or consequence (Metzinger, 2013; Steinert et al., 2019). Assuming that any voluntary motor action relies on the implementation of the agent’s intention(s) (see Pacherie, 2008), we have thus specifically scrutinized active and reactive BCI-mediated actions through the prism of the motor action models we briefly described in the previous section.

Reactive BCI requires the user to intentionally focus his/her attention on external stimuli to potentially control an external device. In such a reactive paradigm, neural modulations are exogenously generated by specific stimulations provided by the BCI system (e.g., Höhne et al., 2011). Thus, EEG-based BCIs rely on visual or auditory evoked potentials such as the P300 event-related potential (ERP) that occurs 300 ms after an important event (see Jin et al., 2012; Lin et al., 2018), the error-related potential (ErrP) that occurs around 200 ms after a mistake (e.g., Dal Seno et al., 2010; Yousefi et al., 2018) or steady state evoked potentials (SSEP) that are induced by oscillating stimuli (e.g., Chen et al., 2017). Interestingly, some of these reactive settings do rely on neural prediction mechanisms. Notably, P300 and ErrP are, respectively, observed when a rare event (i.e., an event inducing “surprise”) or an erroneous effect (i.e., the actual effect was mispredicted) is presented. For instance, reinforcement learning based BCIs, which use neural error responses (e.g., ErrP signals) as reward feedbacks on the agent’s action, offer the potentiality to get autonomous paradigms that would dynamically adapt in case of erroneous classifications (e.g., Marsh et al., 2015; Wirth et al., 2019).

However, in such reactive BCIs, the used brain potentials do not constitute *direct* but rather *retrospective* markers of neural prediction, since they occur *after* the event or the action execution, as the result of the comparison process occurring between the internal model of forthcoming events (prediction) and the actual sensory effect/event (see Wolpert et al., 1995; Hsu et al., 2015).

Counter to reactive BCI, active BCI is *self-paced* and allows the user to endogenously/voluntarily control his/her brain activity—and consequently the external connected device (e.g., robot or computer)—at any time, without being tied to a stimulus (see Rao, 2013). In particular, active BCI mainly relies on motor imagery (MI), which can correspond to a visual or a kinesthetic representation of the motor action (see Neuper et al., 2005). Indeed, MI produces neural activity that is spatiotemporally similar to the activity generated during the actual movement even though it is smaller in amplitude (see Pfurtscheller and Neuper, 1997; Miller et al., 2010). Specifically, MI is linked to recognizable EEG brainwave patterns (see Wierzgała et al., 2018) such as a decrease in the frequency bands μ (8–12 Hz) and β (18–30 Hz) corresponding to Sensorimotor Rhythms (SMR). Notably, SMR decrease occurs in the brain hemisphere that is contralateral to the limb “imagined” movement. One might presume that the use of brain mechanisms that are at play in motor execution should be sufficient to ensure a certain theoretical proximity between MI-BCI mediated actions and natural motor actions. However, the representation of perceptual action goal/outcome remains barely considered in such paradigms, and only a few researchers have investigated the importance of implementing action feedbacks to BCIs (e.g., Quick et al., 2020). From a broader perspective, the mental act that is executed through the MI-BCI is frequently disconnected from the final purpose or the proprioceptive effect of that action (see Beursken, 2013; Jeunet et al., 2016). This could notably explain the high illiteracy rate—corresponding to the inability to control the BCI system—that is commonly observed in participants (see Lee et al., 2019).

Thus, on the one hand, many reactive BCIs are based on *retrospective* markers of sensory prediction occurring *after* a particular event (e.g., P300 or ErrP). On the other hand, most of active BCIs rely on neural activity associated to sensorimotor commands, without considering the importance of the representation of that motor action in terms of its perceptual consequences.

## Toward an Ideomotor Brain-Computer Interface

### Preliminary Experimental Cues

Numerous experiments have highlighted the importance of adding simple sensory and proprioceptive “feedbacks” following the action performed via the BCI, to increase systems’ accuracy and control (e.g., Omar et al., 2010; Suminski et al., 2010; Ramos-Murguialday et al., 2012; Tidoni et al., 2014), or improve users’ experience (e.g., Wang et al., 2019). Such findings fit the *ideomotor view* stating that voluntary action is primarily performed to produce some anticipated or desired effects in the environment (e.g., Le Bars et al., 2019). From this perspective, one might also argue that these sensory feedbacks allow the agents to make perceptual representations of the BCI-mediated action, enhancing the system’s ease of use. In line with this concept, kinesthetic BCIs—where participants have to make an “embodied” representation of the motor action associated with its sensations—have been found to be more efficient than MI-BCI based on external representations of the action (see Neuper et al., 2005).

Importantly, Aflalo et al. (2015) have tested an invasive active BCI based on neural signals related to *prior intentions* occurring *before* their translation into motor commands. They succeeded in identifying the general imagined goal (e.g., picking the glass on the table) from posterior parietal cortex neurons. This interesting paradigm demonstrates that a BCI relying on the neural activity occurring even before motor activations is possible.

Globally, the studies described above support the idea that a BCI based on a perceptual representation of action, earlier than the effective motor command/imagery (active BCI) or the stimulus onset (reactive BCI), could constitute an interesting way to make BCI-mediated action more naturalistic and accurate.

### Decoding Perceptual Intentions

An essential requirement of reactive and active BCIs is to successfully decode the agent’s intention. To this end, reactive BCIs use neural markers relative to attentional processes (SSEP, P300 etc.) while active BCIs systematically rely on motor intentions assessed via modulations in SMR (see Salvaris and Haggard, 2014). However, what if the agent’s intention would also generate identifiable perceptual representations of desired effects in corresponding brain areas (e.g., specific activations in occipital lobes in case of visual representation), as stated by the ideomotor principle? Interestingly, besides the ideomotor field, perceptual activations linked to human intentions, and related to the goal, have already been observed in fMRI studies, notably in the occipital cortex (see Gilbert and Fung, 2018). Neely et al. (2018) also showed that modulations of neural activity in primary visual cortex could be *intentional* (i.e., goal-directed) in rodents.

Then, the ability to decode intentions without using pure motoric activations but rather perceptual representations of desired effects that would be less sophisticated than the complete goal (e.g., Aflalo et al., 2015), appears feasible. Moreover, we believe that such a paradigm would be more appropriate given the disembodied nature of BCI-mediated actions where the peripheral motor system is not supposed to be involved.

### Proposal of a Novel Brain-Computer Interface Paradigm Based on a Century-Old Concept

**FIGURE 2:**
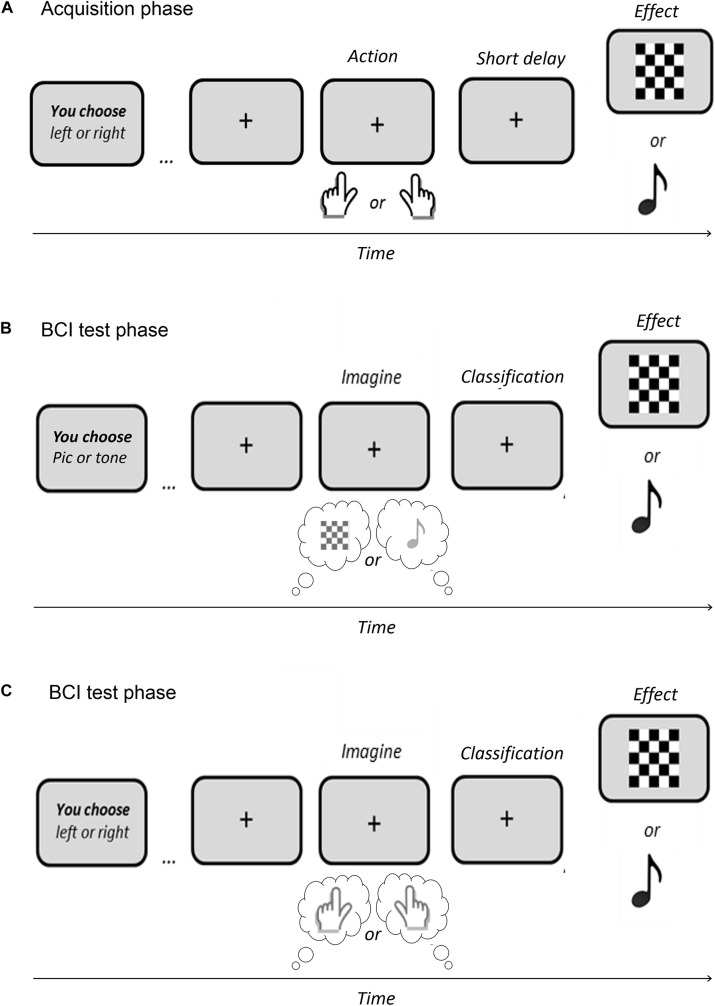
Example of an Ideomotor BCI paradigm. **(A)** Acquisition phase aiming to link simple actions with specific effects (e.g., right key press → visual effect vs. left key press → auditory tone). **(B)** Test phase of a BCI based on the user’s active representation of the effect. **(C)** Test phase of a BCI based on the user’s active representation of the action.

Similarly to ideomotor experiments, an ideomotor BCI paradigm would necessarily require a first acquisition stage, aiming to link each specific action to specific sensory effects. For instance, performing the action A1 would result in the effect E1, while performing the action A2 would generate the effect E2 (see Shin et al., 2010). However, given the low spatial resolution and Signal/Noise Ratio of EEG method, not every sensory effect representation would be appropriate for EEG tracking. Then, properties of sensory feedbacks must be carefully selected to allow the identification of their anticipated representation at a cortical level. Previous experiments have shown that certain visual effects, such as flickering stimuli (see Dignath et al., 2020) or houses and faces (see Hughes and Waszak, 2014), were linked to discernible neural modulations in the occipital and temporal cortex, before their onset (i.e., before the start of the visual stimulation). Moreover, the expectation of auditory effects (i.e., 400-, 600-, and 800-Hz sinusoidal wave tones of 200-ms duration)—normally associated to certain actions—has also been shown to generate an increase of activity in the auditory cortex, in the absence of actual auditory stimulation (see Kühn and Brass, 2010). Using equivalent stimuli (i.e., visual patterns or auditory tones) as action-effects during the acquisition phase (see Figure 2A) might allow the use of *anticipated* neural modulations in corresponding perceptual areas as proxies of *perceptual intentions*.

Interestingly, during this acquisition step, the agent could either perform real motor actions (e.g., pressing a key with a finger) or BCI-mediated actions (e.g., focusing his visual attention on a flickering target or imagining a limb movement) in order to generate the specific effects. We believe that both action types would lead to action-effect binding if a prior intention to act exists (see Caspar et al., 2021). However, intentional binding between actions and effects will certainly be higher in natural motor actions, notably because accuracy and latency are not optimal in BCI systems (see Limerick et al., 2014). For this reason, we suggest to first test the potentiality of an ideomotor BCI with a motoric acquisition phase, as shown in Figure 2A. Then, it would be fascinating to analyze whether simple sensory feedbacks of BCI-mediated actions could also lead to neural modulations in corresponding perceptual areas, before or during the “mental act” (e.g., imagining a limb movement or focusing on a specific flickering target).

Assuming that the mapping between actions and effects would be effective at the end of the acquisition phase, further similar trials would also serve to train and calibrate AI models aiming to decode the “perceptual intention” that should be identifiable *before* action execution (e.g., Waszak et al., 2012; Dignath et al., 2020).

Then, the ability to accurately decode perceptual intentions could lead to two different BCI applications and tasks:

First, as an independent ideomotor BCI where the agent would be instructed to focus on the intended sensory effect, that is, on the perceptual outcome of the action, or more generally on the global action that led to this outcome, as represented in Figures 2B,C, respectively. In the first case, the user’s task (i.e., thinking about the action outcome) should be easier than kinesthetic paradigms that require an embodied representation of the action. More importantly, in both cases, the neural markers used for AI models will be spatially and temporally different from the ones that are currently used in existing BCI paradigms: they should correspond to modulations of activity in the perceptual brain areas involved during action-effect perception, and should be activated *before* the occurrence of the actual outcome or action.

Second, Ideomotor BCI could serve as a hybrid BCI where the user would be instructed to perform another BCI task (MI or Attentional focus) while the decoded perceptual intention would be used in conjunction with other typical neural makers (e.g., SMR or SSVEP), to make the global BCI faster and more accurate. In this situation, the user’s main task will correspond to usual reactive or active BCI paradigms.

In both cases, we believe that the adaptation of BCI paradigms in order to elicit perceptual representations of BCI-mediated actions should improve their technical performance and users’ experience.

## Discussion

In this article, we proposed an experimental framework, in line with the ideomotor principle, to test a new type of non-invasive BCI that would allow to perform more naturalistic BCI-mediated actions.

This perspective depends entirely on the ability to decode efficiently the users’ *perceptual intentions*, by using neural modulations related to action-effect predictions occurring *before* the action (e.g., Kühn et al., 2010; Waszak et al., 2012; Dignath et al., 2020). This point is not trivial for at least two reasons. First, due to the lack of a unified theory regarding the neural patterns underlying action-effect prediction, which could for instance correspond to a *cancelation* (Wolpert et al., 1995), a *preactivation* (Waszak et al., 2012), or a *sharpening* (Yon et al., 2018) of activity in the perceptual units concerned. Second, because the standard method for brain activity recording in non-invasive BCIs, namely EEG, has a low spatial resolution, which prevents from a precise discrimination between different sources of activations in perceptual units. Thus, further studies must harness adequate paradigms to allow perceptual intentions modeling, notably by using sensory feedbacks that would elicit discriminable EEG patterns during action-effect prediction and that would also be discriminable from brain activations resulting from the BCI task itself.

**FIGURE 3:**
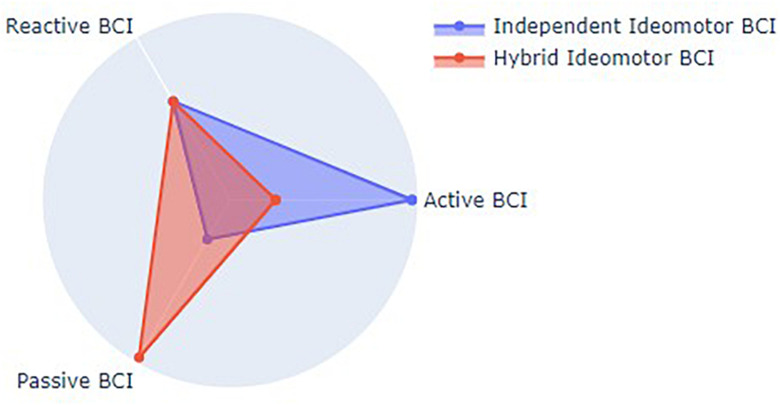
Theoretical positioning of Ideomotor BCI types relative to existing BCI paradigms.

Theoretically, an independent ideomotor BCI would then constitute a novel BCI paradigm located in-between active, reactive and passive paradigms, being endogenously controlled as active paradigms, i.e., the agent would have to think about the perceptual feedback of the BCI-mediated action, but relying on sensory stimuli, i.e., the action-effects, as reactive paradigms. One might also propose to use this ideomotor paradigm in combination with other BCI methods (e.g., MI-BCI). Given the automatic nature of the action-effect prediction process (see Kunde, 2004; Le Bars et al., 2016), the agent would not be instructed to make a conscious representation of the intended feedback, even though EEG markers of action-effect prediction would be used as additional features to improve the BCI accuracy. In this case, the hybrid ideomotor BCI would be closer from passive BCI paradigms but would still rely on sensory stimuli, as represented in Figure 3.

In conclusion, an ideomotor BCI would allow to get closer from natural action by considering the perceptual facet of human action through action-effect prediction. This should improve both users’ experience and systems accuracy. Importantly, an effective BCI paradigm—that would be based on perceptual representation of action would definitely endorse and concomitantly renew the old-fashioned ideomotor theory.

## Data Availability Statement

The original contributions presented in the study are included in the article/supplementary material, further inquiries can be directed to the corresponding author/s.

## Author Contributions

SL and FW developed the idea and the research proposal which were described in the current article. SL wrote the manuscript with supervision from SC and FW. All authors contributed to the article and approved the submitted version.

## Conflict of Interest

The authors declare that the research was conducted in the absence of any commercial or financial relationships that could be construed as a potential conflict of interest.

## Publisher’s Note

All claims expressed in this article are solely those of the authors and do not necessarily represent those of their affiliated organizations, or those of the publisher, the editors and the reviewers. Any product that may be evaluated in this article, or claim that may be made by its manufacturer, is not guaranteed or endorsed by the publisher.

## References

[B1] Aflalo T. Kellis S. Klaes C. Lee B. Shi Y. Pejsa K. (2015). Decoding motor imagery from the posterior parietal cortex of a tetraplegic human. *Science* 348 906–910. 10.1126/science.aaa5417 25999506 PMC4896830

[B2] Baess P. Horváth J. Jacobsen T. Schröger E. (2011). Selective suppression of self-initiated sounds in an auditory stream: an ERP study. *Psychophysiology* 48 1276–1283. 10.1111/j.1469-8986.2011.01196.x 21449953

[B3] Beursken E. S. (2013). *Transparancy in BCI: The Effect of the Mapping Between an Imagined Movement and the Resulting Action on a User’s Sense of Agency*. Bachelor’s thesis, Radboud University, Nijmegen. Available online at: https://theses.ubn.ru.nl/bitstream/handle/123456789/96/Beursken%2C_E.S._1.pdf?sequence=1

[B4] Cardoso-Leite P. Mamassian P. Schütz-Bosbach S. Waszak F. (2010). A new look at sensory attenuation: action-effect anticipation affects sensitivity, not response bias. *Psychol. Sci.* 21 1740–1745. 10.1177/0956797610389187 21119181

[B5] Caspar E. A. De Beir A. Lauwers G. Cleeremans A. Vanderborght B. (2021). How using brain-machine interfaces influences the human sense of agency. *PLoS One* 16:e0245191. 10.1371/journal.pone.0245191 33411838 PMC7790430

[B6] Chen J. Zhang D. Engel A. K. Gong Q. Maye A. (2017). Application of a single-flicker online SSVEP BCI for spatial navigation. *PLoS One* 12:e0178385. 10.1371/journal.pone.0178385 28562624 PMC5451069

[B7] Colzato L. S. Van Wouwe N. C. Hommel B. (2007). Feature binding and affect: emotional modulation of visuo-motor integration. *Neuropsychologia* 45 440–446. 10.1016/j.neuropsychologia.2006.06.032 16926036

[B8] Dal Seno B. Matteucci M. Mainardi L. (2010). Online detection of P300 and error potentials in a BCI speller. *Comput. Intell. Neurosci.* 2010:307254. 10.1155/2010/307254 20169142 PMC2821756

[B9] Desantis A. Roussel C. Waszak F. (2014). The temporal dynamics of the perceptual consequences of action-effect prediction. *Cognition* 132 243–250. 10.1016/j.cognition.2014.04.010 24853627

[B10] Dignath D. Kiesel A. Frings C. Pastötter B. (2020). Electrophysiological evidence for action-effect prediction. *J. Exp. Psychol. Gen.* 149:1148. 10.1037/xge0000707 31750715

[B11] Douibi K. Le Bars S. Lemontey A. Nag L. Balp R. Breda G. (2021). Toward EEG-based BCI applications for industry 4.0: challenges and possible applications. *Front. Hum. Neurosci.* 15:705064. 10.3389/fnhum.2021.705064 34483868 PMC8414547

[B12] Friston K. FitzGerald T. Rigoli F. Schwartenbeck P. Pezzulo G. (2016). Active inference and learning. *Neurosci. Biobehav. Rev.* 68 862–879. 10.1016/j.neubiorev.2016.06.022 27375276 PMC5167251

[B13] Gilbert S. J. Fung H. (2018). Decoding intentions of self and others from fMRI activity patterns. *Neuroimage* 172 278–290. 10.1016/j.neuroimage.2017.12.090 29305161

[B14] Greenwald A. G. (1970). Sensory feedback mechanisms in performance control: with special reference to the ideo-motor mechanism. *Psychol. Rev.* 77:73. 10.1037/h0028689 5454129

[B15] Guger C. Allison B. Z. Großwindhager B. Prückl R. Hintermüller C. Kapeller C. (2012). How many people could use an SSVEP BCI? *Front. Neurosci.* 6:169. 10.3389/fnins.2012.00169 23181009 PMC3500831

[B16] Höhne J. Schreuder M. Blankertz B. Tangermann M. (2011). A novel 9-class auditory ERP paradigm driving a predictive text entry system. *Front. Neurosci.* 5:99. 10.3389/fnins.2011.00099 21909321 PMC3163907

[B17] Hommel B. Müsseler J. Aschersleben G. Prinz W. (2001). The theory of event coding (TEC): a framework for perception and action planning. *Behav. Brain Sci.* 24:849. 10.1017/S0140525X01000103 12239891

[B18] Hsu Y. F. Le Bars S. Hämäläinen J. A. Waszak F. (2015). Distinctive representation of mispredicted and unpredicted prediction errors in human electroencephalography. *J. Neurosci.* 35 14653–14660. 10.1523/JNEUROSCI.2204-15.2015 26511253 PMC6605467

[B19] Hughes G. Waszak F. (2014). Predicting faces and houses: category-specific visual action-effect prediction modulates late stages of sensory processing. *Neuropsychologia* 61 11–18. 10.1016/j.neuropsychologia.2014.06.002 24930591

[B20] James W. (1890). *The Principles Of Psychology*, Vol. I, II. Cambridge, MA: Harvard University Press. 10.1037/10538-000

[B21] Jeunet C. Jahanpour E. Lotte F. (2016). Why standard brain-computer interface (BCI) training protocols should be changed: an experimental study. *J. Neural Eng.* 13:036024. 10.1088/1741-2560/13/3/036024 27172246

[B22] Jin J. Allison B. Z. Kaufmann T. Kübler A. Zhang Y. Wang X. (2012). The changing face of P300 BCIs: a comparison of stimulus changes in a P300 BCI involving faces, emotion, and movement. *PLoS One* 7:e49688. 10.1371/journal.pone.0049688 23189154 PMC3506655

[B23] Klaffehn A. L. Baess P. Kunde W. Pfister R. (2019). Sensory attenuation prevails when controlling for temporal predictability of self-and externally generated tones. *Neuropsychologia* 132:107145. 10.1016/j.neuropsychologia.2019.107145 31319119

[B24] Kögel J. Schmid J. R. Jox R. J. Friedrich O. (2019). Using brain-computer interfaces: a scoping review of studies employing social research methods. *BMC Med. Ethics* 20:18. 10.1186/s12910-019-0354-1 30845952 PMC6407281

[B25] Kok P. Jehee J. F. De Lange F. P. (2012). Less is more: expectation sharpens representations in the primary visual cortex. *Neuron* 75 265–270. 10.1016/j.neuron.2012.04.034 22841311

[B26] Kühn S. Brass M. (2010). Planning not to do something: does intending not to do something activate associated sensory consequences? *Cogn. Affect. Behav. Neurosci.* 10 454–459. 10.3758/CABN.10.4.454 21098806

[B27] Kühn S. Keizer A. W. Rombouts S. A. Hommel B. (2011). The functional and neural mechanism of action preparation: roles of EBA and FFA in voluntary action control. *J. Cogn. Neurosci.* 23 214–220. 10.1162/jocn.2010.21418 20044885

[B28] Kühn S. Seurinck R. Fias W. Waszak F. (2010). The internal anticipation of sensory action effects: when action induces FFA and PPA activity. *Front. Hum. Neurosci.* 4:54. 10.1016/j.neuropsychologia.2014.06.002 20661462 PMC2907885

[B29] Kunde W. (2004). Response priming by supraliminal and subliminal action effects. *Psychol. Res.* 68 91–96. 10.1007/s00426-003-0147-4 14634809

[B30] Le Bars S. Darriba Á Waszak F. (2019). Event-related brain potentials to self-triggered tones: impact of action type and impulsivity traits. *Neuropsychologia* 125 14–22. 10.1016/j.neuropsychologia.2019.01.012 30685504

[B31] Le Bars S. Hsu Y. F. Waszak F. (2016). The impact of subliminal effect images in voluntary vs. stimulus-driven actions. *Cognition* 156 6–15. 10.1016/j.cognition.2016.07.005 27467892

[B32] Lee M. H. Kwon O. Y. Kim Y. J. Kim H. K. Lee Y. E. Williamson J. (2019). EEG dataset and OpenBMI toolbox for three BCI paradigms: an investigation into BCI illiteracy. *Gigascience* 8:giz002. 10.1093/gigascience/giz002 30698704 PMC6501944

[B33] Limerick H. Coyle D. Moore J. W. (2014). The experience of agency in human-computer interactions: a review. *Front. Hum. Neurosci.* 8:643. 10.3389/fnhum.2014.00643 25191256 PMC4140386

[B34] Lin Z. Zhang C. Zeng Y. Tong L. Yan B. (2018). A novel P300 BCI speller based on the Triple RSVP paradigm. *Sci. Rep.* 8:3350. 10.1038/s41598-018-21717-y 29463870 PMC5820322

[B35] Marsh B. T. Tarigoppula V. S. A. Chen C. Francis J. T. (2015). Toward an autonomous brain machine interface: integrating sensorimotor reward modulation and reinforcement learning. *J. Neurosci.* 35 7374–7387. 10.1523/JNEUROSCI.1802-14.2015 25972167 PMC6705437

[B36] Metzinger T. K. (2013). The myth of cognitive agency: subpersonal thinking as a cyclically recurring loss of mental autonomy. *Front. Psychol.* 4:931. 10.3389/fpsyg.2013.00931 24427144 PMC3868016

[B37] Miller K. J. Schalk G. Fetz E. E. den Nijs M. Ojemann J. G. Rao R. P. (2010). Cortical activity during motor execution, motor imagery, and imagery-based online feedback. *Proc. Natl. Acad. Sci. U.S.A.* 107 4430–4435. 10.1073/pnas.0913697107 20160084 PMC2840149

[B38] Muhle-Karbe P. S. Krebs R. M. (2012). On the influence of reward on action-effect binding. *Front. Psychol.* 3:450. 10.3389/fpsyg.2012.00450 23130005 PMC3487417

[B39] Neely R. M. Koralek A. C. Athalye V. R. Costa R. M. Carmena J. M. (2018). Volitional modulation of primary visual cortex activity requires the basal ganglia. *Neuron* 97 1356–1368. 10.1016/j.neuron.2018.01.051 29503189

[B40] Neuper C. Scherer R. Reiner M. Pfurtscheller G. (2005). Imagery of motor actions: differential effects of kinesthetic and visual–motor mode of imagery in single-trial EEG. *Cogn. Brain Res.* 25 668–677. 10.1016/j.cogbrainres.2005.08.014 16236487

[B41] Omar C. Akce A. Johnson M. Bretl T. Ma R. Maclin E. (2010). A feedback information-theoretic approach to the design of brain–computer interfaces. *Int. J. Hum. Comput. Int.* 27 5–23. 10.1080/10447318.2011.535749

[B42] Pacherie E. (2008). The phenomenology of action: a conceptual framework. *Cognition* 107 179–217. 10.1016/j.cognition.2007.09.003 17950720

[B43] Paelecke M. Kunde W. (2007). Action-effect codes in and before the central bottleneck: evidence from the psychological refractory period paradigm. *J. Exp. Psychol. Hum. Percept. Perform.* 33:627. 10.1037/0096-1523.33.3.627 17563226

[B44] Pfurtscheller G. Neuper C. (1997). Motor imagery activates primary sensorimotor area in humans. *Neurosci. Lett.* 239 65–68. 9469657 10.1016/s0304-3940(97)00889-6

[B45] Prinz W. (1997). Perception and action planning. *Eur. J. Cogn. Psychol.* 9 129–154. 10.1080/713752551

[B46] Quick K. M. Weiss J. M. Clemente F. Gaunt R. A. Collinger J. L. (2020). “Intracortical microstimulation feedback improves grasp force accuracy in a human using a brain-computer interface^∗^,” in *Proceedings of the 2020 42nd Annual International Conference of the IEEE Engineering in Medicine Biology Society (EMBC)*, Piscataway, NJ: IEEE, 3355–3358. 10.1109/EMBC44109.2020.9175926 PMC7717497 33018723

[B47] Rainey S. Maslen H. Savulescu J. (2020). When thinking is doing: responsibility for BCI-Mediated action. *AJOB Neurosci.* 11 46–58. 10.1080/21507740.2019.1704918 32009590 PMC7034530

[B48] Ramos-Murguialday A. Schürholz M. Caggiano V. Wildgruber M. Caria A. Hammer E. M. (2012). Proprioceptive feedback and brain computer interface (BCI) based neuroprostheses. *PLoS One* 7:e47048. 10.1371/journal.pone.0047048 23071707 PMC3465309

[B49] Rao R. P. (2013). *Brain-Computer Interfacing: An Introduction.* Cambridge, MA: Cambridge University Press. 10.1017/CBO9781139032803

[B50] Roussel C. Hughes G. Waszak F. (2013). A preactivation account of sensory attenuation. *Neuropsychologia* 51 922–929. 10.1016/j.neuropsychologia.2013.02.005 23428377

[B51] Roussel C. Hughes G. Waszak F. (2014). Action prediction modulates both neurophysiological and psychophysical indices of sensory attenuation. *Front. Hum. Neurosci.* 8:115. 10.3389/fnhum.2014.00115 24616691 PMC3937955

[B52] Salvaris M. Haggard P. (2014). Decoding intention at sensorimotor timescales. *PLoS One* 9:e85100. 10.1371/journal.pone.0085100 24523855 PMC3921113

[B53] Shin Y. K. Proctor R. W. Capaldi E. J. (2010). A review of contemporary ideomotor theory. *Psychol. Bull.* 136:943. 10.1037/a0020541 20822210

[B54] Stanley J. Gowen E. Miall R. C. (2007). Effects of agency on movement interference during observation of a moving dot stimulus. *J. Exp. Psychol. Hum. Percept. Perform.* 33:915. 10.1037/0096-1523.33.4.915 17683237 PMC3073012

[B55] Steinert S. Bublitz C. Jox R. Friedrich O. (2019). Doing things with thoughts: brain-computer interfaces and disembodied agency. *Philos. Technol.* 32 457–482. 10.1007/s13347-018-0308-4

[B56] Stock A. Stock C. (2004). A short history of ideo-motor action. *Psychol. Res.* 68 176–188. 10.1007/s00426-003-0154-5 14685855

[B57] Suminski A. J. Tkach D. C. Fagg A. H. Hatsopoulos N. G. (2010). Incorporating feedback from multiple sensory modalities enhances brain–machine interface control. *J. Neurosci.* 30 16777–16787. 10.1523/JNEUROSCI.3967-10.2010 21159949 PMC3046069

[B58] Tidoni E. Gergondet P. Kheddar A. Aglioti S. M. (2014). Audio-visual feedback improves the BCI performance in the navigational control of a humanoid robot. *Front. Neurorobot.* 8:20. 10.3389/fnbot.2014.00020 24987350 PMC4060053

[B59] Wang W. Liu Y. Li Z. Wang Z. He F. Ming D. Yang D. (2019). “Building multi-modal sensory feedback pathways for SRL with the aim of sensory enhancement via BCI,” in *IEEE International Conference on Robotics and Biomimetics* (Sanya: Robio), 2439–2444.

[B60] Waszak F. Cardoso-Leite P. Hughes G. (2012). Action effect anticipation: neurophysiological basis and functional consequences. *Neurosci. Biobehav. Rev.* 36 943–959. 10.1016/j.neubiorev.2011.11.004 22108008

[B61] Wierzgała P. Zapała D. Wojcik G. M. Masiak J. (2018). Most popular signal processing methods in motor-imagery BCI: a review and meta-analysis. *Front. Neuroinform.* 12:78. 10.3389/fninf.2018.00078 30459588 PMC6232268

[B62] Wirth C. Dockree P. M. Harty S. Lacey E. Arvaneh M. (2019). Towards error categorisation in BCI: single-trial EEG classification between different errors. *J. Neural Eng.* 17:016008. 10.1088/1741-2552/ab53fe 31683267

[B63] Wolpert D. M. Ghahramani Z. Jordan M. I. (1995). An internal model for sensorimotor integration. *Science* 269 1880–1882. 7569931 10.1126/science.7569931

[B64] Yon D. Gilbert S. J. de Lange F. P. Press C. (2018). Action sharpens sensory representations of expected outcomes. *Nat. Commun.* 9 1–8. 30327503 10.1038/s41467-018-06752-7 PMC6191413

[B65] Yousefi R. Sereshkeh A. R. Chau T. (2018). Exploiting error-related potentials in cognitive task based BCI. *Biomed. Phys. Eng. Express* 5:015023.

[B66] Zander T. O. Krol L. R. (2017). Team PhyPA: brain-computer interfacing for everyday human-computer interaction. *Period. Polytech. Electr. Eng. Comput. Sci.* 61 209–216. 10.3311/PPee.10435

[B67] Ziessler M. Nattkemper D. (2011). The temporal dynamics of effect anticipation in course of action planning. *Q. J. Exp. Psychol.* 64 1305–1326. 10.1080/17470218.2011.553067 21416456

